# Smart Textile Impact Sensor for e-Helmet to Measure Head Injury

**DOI:** 10.3390/s24092919

**Published:** 2024-05-03

**Authors:** Manob Jyoti Saikia, Arar Salim Alkhader

**Affiliations:** 1 Department of Electrical Engineering, University of North Florida, Jacksonville, FL 32224, USA; 2 Department of Electrical, Computer, and Biomedical Engineering, University of Rhode Island, Kingston, RI 02881, USA

**Keywords:** concussion, e-helmet, e-textile, head impact, head injury, mTBI, sensor, smart textile, smart fabric

## Abstract

Concussions, a prevalent public health concern in the United States, often result from mild traumatic brain injuries (mTBI), notably in sports such as American football. There is limited exploration of smart-textile-based sensors for measuring the head impacts associated with concussions in sports and recreational activities. In this paper, we describe the development and construction of a smart textile impact sensor (STIS) and validate STIS functionality under high magnitude impacts. This STIS can be inserted into helmet cushioning to determine head impact force. The designed 2 × 2 STIS matrix is composed of a number of material layered structures, with a sensing surface made of semiconducting polymer composite (SPC). The SPC dimension was modified in the design iteration to increase sensor range, responsiveness, and linearity. This was to be applicable in high impact situations. A microcontroller board with a biasing circuit was used to interface the STIS and read the sensor’s response. A pendulum test setup was constructed to evaluate various STISs with impact forces. A camera and Tracker software were used to monitor the pendulum swing. The impact forces were calculated by measuring the pendulum bob’s velocity and acceleration. The performance of the various STISs was measured in terms of voltage due to impact force, with forces varying from 180 to 722 N. Through data analysis, the threshold impact forces in the linear range were determined. Through an analysis of linear regression, the sensors’ sensitivity was assessed. Also, a simplified model was developed to measure the force distribution in the 2 × 2 STIS areas from the measured voltages. The results showed that improving the SPC thickness could obtain improved sensor behavior. However, for impacts that exceeded the threshold, the suggested sensor did not respond by reflecting the actual impact forces, but it gave helpful information about the impact distribution on the sensor regardless of the accurate expected linear response. Results showed that the proposed STIS performs satisfactorily within a range and has the potential to be used in the development of an e-helmet with a large STIS matrix that could cover the whole head within the e-helmet. This work also encourages future research, especially on the structure of the sensor that could withstand impacts which in turn could improve the overall range and performance and would accurately measure the impact in concussion-causing impact ranges.

## 1. Introduction

Concussion is a form of brain injury resulting from impacts to the head or swift back-and-forth head movements and causing alterations in brain tissue shape and harm to brain cells. Given the brain’s pivotal role as the body’s control center, any cell damage is significant and has profound consequences. The Center for Disease Control (CDC) reported annual concussion cases of approximately 1.7 to 3.8 million in the United States [1]. While a concussion is typically not fatal, it can significantly alter one’s life. The damage to brain cells induces metabolic and chemical shifts, leading to communication and functional challenges that necessitate substantial concussion treatment. Numerous criteria for identifying injuries have been proposed in the literature, often rooted in established thresholds for brain injuries [2,3]. The Wayne State Tolerance Curve (WSTC) serves as a gauge for the threshold of tolerance in the human head, pinpointing the limit at which skull fractures become an equivalent indicator of tolerance for brain injuries [4]. Gadd [2] introduced the Severity Index (SI), incorporating linear acceleration as a measure to indicate both the severity and the cause of injuries. The US National Highway Traffic Safety Administration (NHTSA) proposed the Head Impact Criteria (HIC) as a substitute for the Severity Index (SI) [3]. The SI is currently extensively employed for traumatic brain injury (TBI) diagnosis, relying on linear head acceleration. The Abbreviated Injury Scale (AIS) is a classification system that anatomically gauges severity of injury on a scale of 0 to 6, where 0 signifies no injury, and 6 denotes an injury that is fatal [5].

**Figure 1:**
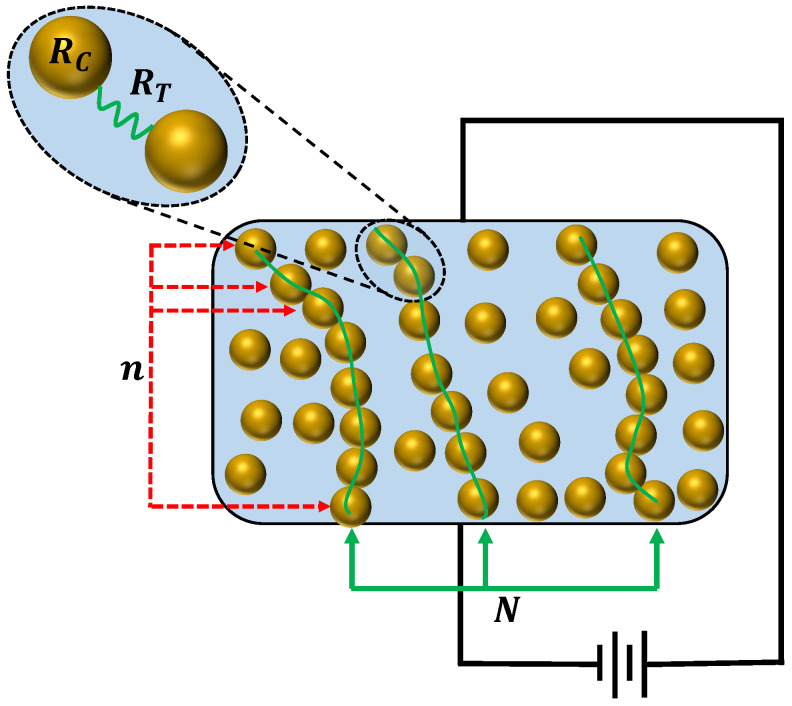
Resistivity of semiconducting polymer composite (SPC).

The modern e-Helmet uses accelerometers and gyroscopes as impact sensors for concussion detection. There are some research studies on accelerometers and gyroscopes for concussion. However, smart textile sensors are seldom used to measure head impact. There are some studies on smart textiles for presser sensing. Most of the smart textile studies are limited to low-level force or pressure testing performed on bench tops. However, smart textiles are potentially suitable for constructing an e-Helmet along with other conventional electronics to measure head impacts. This paper introduces a smart textile impact sensor (STIS) and examines the general performance of STIS under high-impact forces. This STIS can be integrated into the cushion of a helmet to estimate the impact force on the head. The designed 2 × 2 STIS matrix consists of different layers of various materials, including a sensing element based on semiconducting polymer composite (SPC) material [Figure 1]. The thickness of the SPC was increased subsequently in the design phase to increase the range of the sensor to be applicable in high-impact situations.

An experimental test setup with a pendulum was constructed to evaluate various STISs with impact forces. Tracker software was utilized to monitor the pendulum mass with a special camera. The acceleration and speed of the pendulum mass were calculated, and thereby the impact forces were estimated. As a result of the impact forces, the voltage response of various STISs was measured, with impact forces ranging from 180 to 722 N. The threshold impact force at which the sensors behave non-linearly was determined through data analysis. Analyses of linear regression were carried out in order to assess the sensitivity of the sensors to linear regions. Additionally, voltage measurements were used to estimate the impact force distribution in the 2 × 2 STIS areas using a simplified model. The results showed that by thickening the SCPS, a better sensor behavior could be obtained. However, at extremely high impacts (beyond threshold points), the proposed sensor with SPC did not provide readings that were indicative of the true applied impact forces. However, it provided useful data that illustrated the impact distribution through the measurement of voltages. However, in a limited range, the STIS performed as expected and may be useful for the future creation of an e-helmet with a STIS matrix that could cover the whole head within the e-helmet.

## 2. Available e-Helmet Technologies

Numerous commercially available tools facilitate the detection and measurement of concussions. This section provides an overview of the primary technologies employed in these devices. Typically, accelerometers are utilized to gauge the frequency of impacts and monitor the forces exerted on the head. Acceleration is measured in either meters per second squared ($m/s^{2}$) or G-force (g), with 1 g equaling the standard 9.8 $m/s^{2}$ gravitational acceleration on Earth. Thus, accelerations can be measured for constant gravity or due to dynamic impact forces involving movement, often along one, two, or multiple axes. Presently, the three-axis accelerometers are the most prevalent choice. These accelerometers offer advantages such as their compact size and real-time monitoring of impact data, as demonstrated in applications such as tracking football players during a game [6]. Gyroscopes, in addition to linear accelerometers, are mainly employed to calculate angular acceleration. Many accelerometers function by detecting capacitance changes, where alterations in the capacitance of internal plates connected by springs reflect movement-induced changes in acceleration. Others utilize the piezoelectric effect, generating electrical charge outputs in response to mechanical forces. Accelerometers often have impact-force measurement ranges from ±1 g up to ±250 g, with lower ranges providing more resolution and better sensitivity to readings. An example of accelerometer application is found in the Riddell InSite Analytics smart helmet technology, where accelerometers are embedded in the helmet, triggering alerts when impacts surpass a predefined threshold [7].

The Riddell sideline system comprises six single-axis accelerometers integrated into a helmet, triggering alerts upon impact surpassing a preset threshold. The Riddell Insight features a five-zone sensor pad within the helmet [7,8]. Numerous other devices such as the Impact Assessment System, GForce Tracker, SIM-P (individual) and SIM-G (team), BodiTrak Head Health Network, and Shockbox by i1 Biometrics, as well as the Vector, X-Patch, Reebok Checklight, and X-Guard by X2 Biosystems, offer diverse models and configurations, generally employing three-axis accelerometers and gyroscopes at varied positions. Gyroscopes are commonly situated near the mouthguard, while accelerometers are positioned behind the ear, on headbands, or within skull caps. Table 1 lists the available technologies. Accelerometers and gyroscopes are the primary sensors utilized in concussion-detection devices, while smart textiles, although not prevalent, could emerge as potential alternatives or complements to existing technologies.

## 3. Background of Smart Textiles

Smart textiles, also referred to as electronic textiles, are specialized fabric structures designed or modified to perform specific functions such as detecting and sensing signals. Their appeal lies in their simplicity and wearability, making them versatile for applications, particularly in bio-signal sensing and biomedical uses [9,10,11]. The concept of smart textiles involves modifying the substrates of certain textiles extrinsically or intrinsically to imbue them with sensing functionality when interacting with users or their surroundings [10,11]. Textiles with sensing capabilities are termed smart textile sensors (STSs) and capable of detecting variables such as temperature, pressure, force, electrical current, humidity, and chemicals. STSs fall under the category of smart textile transducers, fabrics altered to function as sensors, actuators, or energy storage and harvesting systems. While STSs can serve various advanced functions, the primary categories include temperature, force, humidity, current, pressure, and chemical sensing.

Existing literature classifies the utility of smart textiles into two primary categories: ornamental and functional. Ornamental smart textiles find widespread use in commercial applications, such as generating light or changing colors in response to pressure, force, vibrations, heat, or sound. Embedded electronics within the textile can power and control these processes, including the adjustment of light intensity. Functional smart textiles are predominantly employed in wearable contexts for sports, medical purposes [12], aerospace applications [13], and military uses to provide protective measures against external hazards or improve the user’s performance [10]. Examples of smart textile functionalities encompass regulating body temperature, releasing drugs, controlling muscle contractions, offering radiation protection, and supporting space travel.

While some materials and designs have been proposed for smart textiles, there are currently no standardized elements or established methods for constructing smart textile sensors (STSs) due to the field being in its developmental phase. While incorporating smart textiles into portable and wearable devices is advantageous, building complete systems solely with smart textiles, without other electronics, remains challenging. Combining both conventional electronics and smart textiles, along with ongoing advancements in textile sensing elements, method for designing, and building methods, could yield more sophisticated and integrated sensing systems. Enhancing and setting standards for smart textile sensors necessitates thorough studies of their characteristics through reliable validation methods, including both bench tests and in-field testing. Although the behavior of certain commercial flexible smart textile sensors has been explored, there is a scarcity of research on the performance of impact sensors made from semiconducting polymer composite as a sensing material for STS, especially in the context of high-impact forces.

In this section, we describe the development of smart textile impact sensor (STIS) using semiconducting polymer composite material.

### 3.1. Smart Textile Impact Sensor (STIS)

Semiconducting polymer composites (SPCs) are a commonly found piezoresistivity-based force or impact-sensing material [14,15,16,17,18,19,20]. In essence, the value of these composites for such applications lies in their ability to undergo changes in electrical resistivity when subjected to forces. This attribute is a result of their structure, comprising a matrix of nonconductive material infused with randomly dispersed nanoparticle fillers made of conductive material. Unlike fiber reinforcements, the microstructure of such composites is characterized by random whisker reinforcement [13,21]. Upon application of an impact force to a semiconducting polymer composite, the gap between the conductive particles shifts, consequently altering the overall electrical resistance of the composite [19]. This alteration in electrical resistivity is due to the strain induced by the applied impact force, causing an alternation in the material’s band structure. This, in turn, results in varying excitation levels of electrons into the conduction band, leading to fluctuations in the density of current carriers and ultimately altering the electrical resistivity of the material.

When subjected to an impacting force, specifically compressive stress, the composite structure experiences a phenomenon known as Brownian motion [14], causing the conductive filler particles suspended in the matrix to randomly proceed nearer one another. This matrix strain leads to alterations in the initial electrical resistivity of the semiconducting polymer material. The change in resistivity is governed by two primary types of resistances: constriction resistance and tunneling resistance. Constriction resistance pertains to the individual conductive element (a single conducting spot) within the filler, and tunneling resistance involves two adjacent conducting elements. The tunnels formed between these adjacent filler particles serve as conduits for current flow during conduction, and this current is referred to as tunneling current [22]. The aggregate SPC resistance (R) can be expressed as in [19,21]:

(1) $$R=\frac{\left(n-1\right)R_{T}+nR_{c}}{N}$$

here, $R_{c}$ represents the constriction resistance, $R_{T}$ denotes the tunneling resistance, *n* signifies the count of conductive filler elements forming a single conducting way, and *N* indicates the overall number of effective conducting ways.

**Figure 2:**
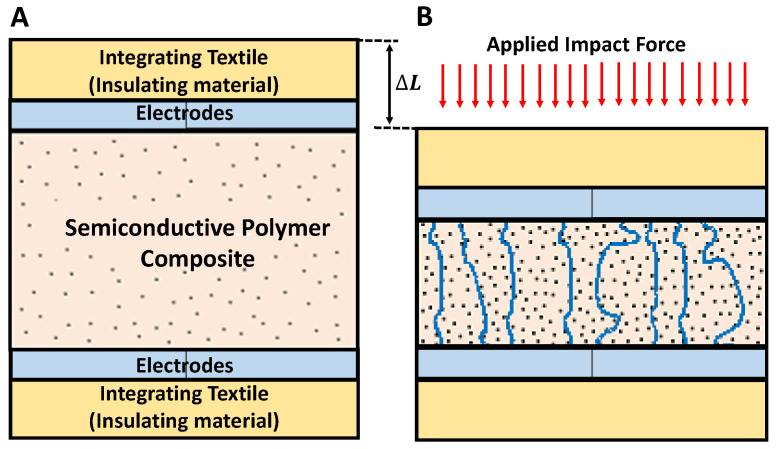
Semiconducting- polymer-composite (SPC)-based smart textile sensor for impact sensing: (**A**) prior to application of an impact force and (**B**) subjected to an impact force.

The main focus of this work is on the development of a semiconductive-polymer-composite (SPC)-material-based smart textile impact sensor (STIS) for high-impact sensing similar to the real-life head impact on the American football field. Our STIS is constructed with an SPC layer placed between two insulating layers, and two layers of electrodes are inserted, as depicted in Figure 2A. When an impact force is applied to the sensor’s surface, the overall electrical resistivity decreases due to a reduction in the resistivity of the SPC, as indicated in Figure 2, and a slight decrease in the electrode–SPC interfacing resistance. This occurs because the applied impact force diminishes the separation between the conductive filler elements in the polymer matrix, forming an increased number of conductive ways, as illustrated in Figure 2B.

### 3.2. Design

Smart textile sensors are crafted from suitable smart textiles, and adding sensing functionality requires modifications. To transform regular textiles into smart textiles with desired sensing characteristics, intrinsic or extrinsic adjustments are essential. Most smart textile sensors (STSs) comprise three key components: sensing materials, conductors, and the insulator and integrating textile [10,21]. While sensors based on STSs are well-suited for detecting low-impact events such as finger pressing, they may exhibit instability and nonlinearity in high-impact ranges. Therefore, the design of STS-based impact sensors must be optimized for applications requiring sensing in the high-impact range, such as those associated with concussion detection.

**Figure 3:**
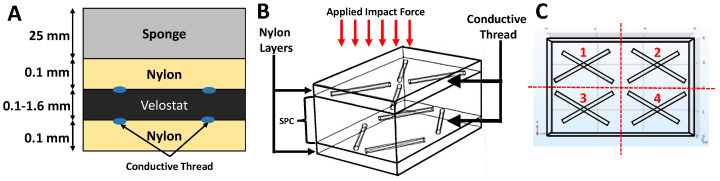
Smart textile impact sensor (STIS): (**A**) layered design, (**B**) 3-D visualization, and (**C**) four areas of sensing.

In this study, an iterative design approach was employed to manually sew smart textile impact sensors (STISs) from smart textiles, integrating them into the helmet cushion. Each iteration involved laboratory testing using a high-impact application setup to analyze sensing behavior. Subsequent improvements were made by modifying the three STIS layers, particularly by modifying the thickness of the SPC sensing element. The sensor comprises top and bottom layers of insulating textiles, while the middle layer accommodates sensing and conduction materials. Additionally, a sponge layer was introduced on top of the sensor to directly interact with the applied impact force, as illustrated in Figure 3. The middle layer incorporates a carbon-filled conducting polyethylene film known as Velostat [23].

We varied the thickness of the sensing element, based on Velostat, in different designs, recognizing its impact on the sensor’s high-impact sensing capability. This paper presents sensors with sensing element thicknesses of 0.2 mm and 0.4 mm, with the final sensor having a 1.6 mm thickness. The two integrating textile layers utilized a 100% nylon plain weave. Nylon, a widely used elastic synthetic fiber, is known for its durability, excellent weather and abrasion resistance, and a volume resistivity of approximately $10^{16}$ Ohm/cm.

**Figure 4:**
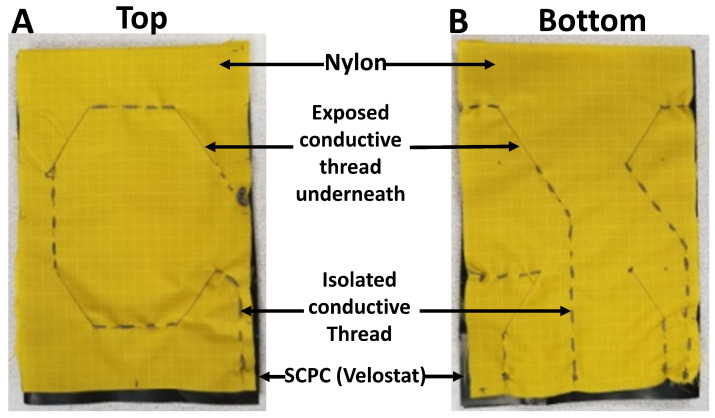
Constructed smart textile impact sensor (STIS): (**A**) top and (**B**) bottom view.

The overall sensor surface area measures 24 ${\mathrm{cm}}^{2}$, divided into four $3\times 2=6\;{\mathrm{cm}}^{2}$ sensing areas, located on each side, as illustrated in Figure 3C. Each individual area operates autonomously and provides feedback regarding the impact magnitude through its respective electrodes. These subdivided sensors collectively form a 2×2 sensor matrix. A conductive thread is hand-stitched to establish the circuit in the sensor. The conducting threads are stitched onto the nylon material to establish direct contact with the Velostat layer at sensing areas from both the top and bottom, as depicted in Figure 3 and Figure 4. A continuously drawn two-ply 316L stainless steel serves as the conductive thread, possessing a resistivity of 1.29 Ohm/inch.

The sensor surfaces and conductive thread collectively induce a piezo-resistive effect when subjected to impact forces. The impact force exerted on the sensing areas is transformed into voltage variations throughout the sensor areas, resulting from a resistance change within an electrical sensing circuit. Each distinct sensing area is connected at its individual point and possesses a distinct connection point. Utilizing resistor-based circuits, voltage divider circuits were established for each of the four sensors areas, which were connected to a microcontroller board for data acquisition. The impact force (*F*) was measured in terms of the change in voltage (δV), where F∝δV, through the use of a voltage divider circuit.

## 4. Experimental Study

**Figure 5:**
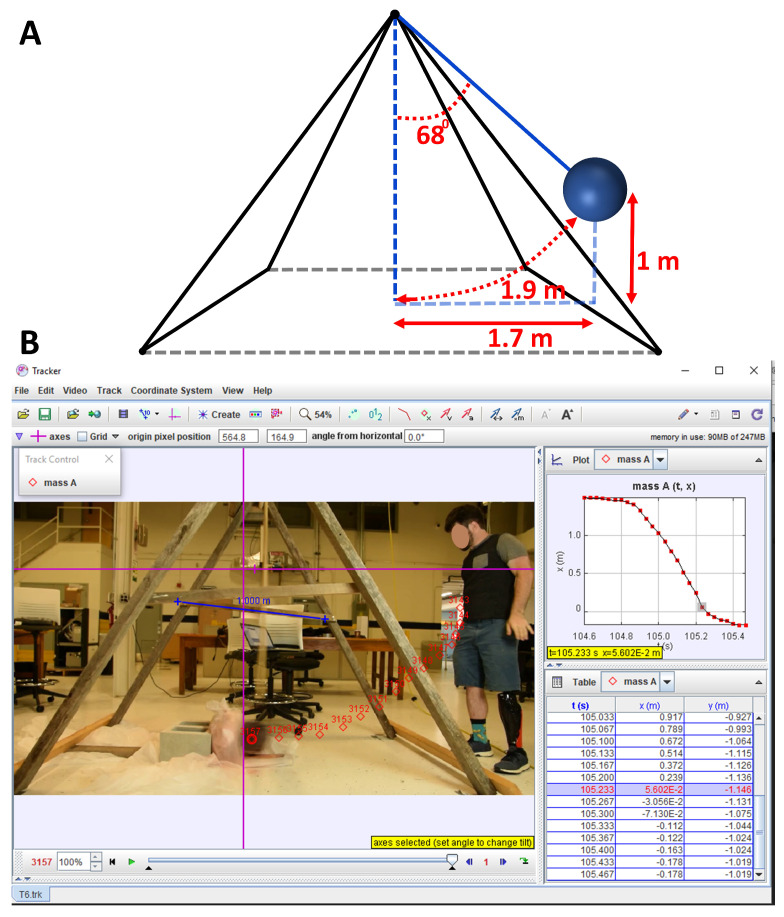
(**A**) Description of the pendulum setup’s dimensions and (**B**) the tracking system of the pendulum.

To assess various sensor designs, an experimental test setup simulating impacts was created. For mimicking real-life head-impact scenarios, a pendulum setup was constructed with a 1.6 m arm and a 17 kg mass attached at the bottom, as depicted in Figure 5A. The smart textile impact sensor (STIS) was affixed to a sandbag, stabilized by a rigid concrete block at the pendulum’s base. The sandbag replicated the curvature of an electronic helmet. It is important to note that the attachment setup between the sensor and the sandbag was not entirely rigid, ensuring that the applied impact was not fully transferred to the sensor. This testing setup resembled electronic helmet construction and testing. The impact intensity was regulated by adjusting the height from which the weight (pendulum mass) was released and by adding an additional push during release. Using a software called Tracker, the pendulum’s velocity and acceleration were measured to estimate the applied impact force, as illustrated in Figure 5B.

The microcontroller board recorded the sensor’s response in terms of voltage change (δV). Three different smart textile impact sensors (STISs) were tested, each designed with varying sensing element thicknesses (0.2 mm, 0.4 mm, and 1.6 mm). In each test, a 17 kg mass was dropped approximately 1 m above and 1.7 m horizontally from the bottom of the pendulum, with the pendulum arm positioned at 68 degrees, as depicted in Figure 5A. Additionally, the experimenter applied an extra force by pushing the mass at the initial release position during each test. The distance covered by the massive mass of the pendulum in each test was determined using the arc distance Formula (2), given by the following:

(2) Arcdistance=2πrθ/360

where r=1.6 m is the pendulum arm length and for $\theta =68^{\circ }$, denotes the angle of the pendulum arm from the rest point, with the *arc distance* being 1.9 m.

## 5. Data Collection

**Figure 6:**
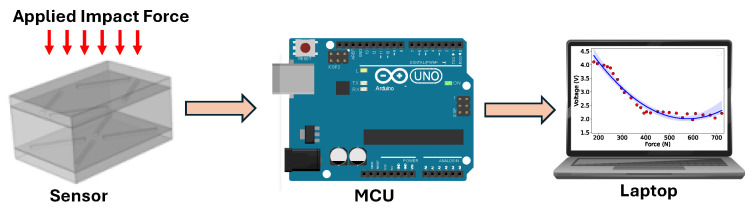
Main component of the sensing system.

The STISs were interfaced with the ADC of the microcontroller board and connected to a laptop computer using a USB cable [Figure 6]. Voltage measurements corresponding to different levels of impact forces were conducted separately for the three STISs with a sensing element thicknesses of 0.2 mm, 0.4 mm, and 1.6 mm. Simultaneous measurements were taken for all four sensing areas of each STIS, as they were linked to four input channels (A1–A3) of the ADC. The measurements reflected the magnitude of the impact force applied to the four areas. The tests involved varying the impact forces generated by external impacts on the pendulum during its release. Momentum, kinetic energy, and acceleration were measured for each impact test. The impact force on the sensor was determined by multiplying mass with acceleration, and the applied impact was calculated by dividing the exerted impact force by the sensor’s surface area ($0.0024\;m^{2}$). The overall voltage reading from the four sensing areas was obtained by summing the voltage responses from each area. Data analysis was conducted using MatLab. Given that the STISs formed a 4 × 4 matrix, the voltage distribution was utilized to compute the impact force distribution across the four sensing areas for analytical purposes.

## 6. Results

**Figure 7:**
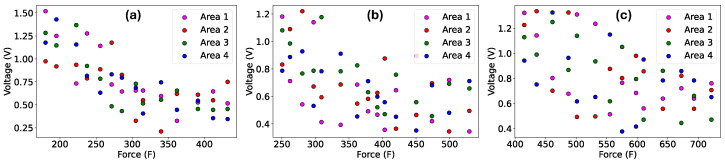
Applied impact forces vs. the voltage measurements on the smart textile impact sensor with (**a**) 0.2 mm, (**b**) 0.4 mm, and (**c**) 1.6 mm sensing element.

The scatter plots in Figure 7a–c depict the voltage measurements at each sensor area for the three STISs against the applied impact forces during various impact tests. The figure additionally illustrates the voltage distribution across the four sensor areas. The voltage decreased with the increase in applied impact forces in all the sensor areas. We also noticed that for low-impact forces, the voltage measurements were less concentrated in each of the four sensing areas for all three STISs than for high-level impact forces. Overall, this suggests that the spatial resolution is better for low-level impact forces. Discerning the relationship between the applied impact force and the voltage readings in each individual area was challenging, as indicated in Figure 7. This difficulty arose due to the precise impact location within the 4 × 4 sensor matrix remaining unknown. The impact force was also not concentrated in the same area, and hence the impact force distribution in the four areas were not known. In order to find a force and voltage relationship, the individual voltage measurements in each area were added together.

**Figure 8:**
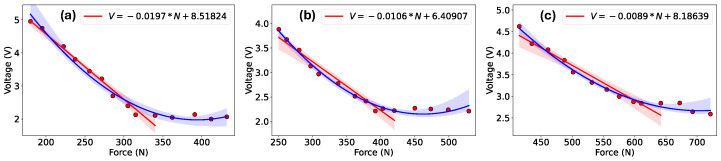
Applied impact forces vs. the total voltages of the smart textile impact sensor with (**a**) 0.2 mm, (**b**) 0.4 mm, and (**c**) 1.6 mm sensing element.

In Figure 8a–c, the total voltage is plotted against the overall applied impact force for the three sensors. Second-order polynomial regression lines (depicted in blue) with a 95% confidence interval were employed to model the data points. It can be observed from Figure 8a,b that the 0.2 mm and 0.4 mm sensors exhibited a linear relationship between force and voltage up to approximately 340 N and 420 N, respectively. However, for the high-impact forces, the sensors were saturated. Hence, we increased the thickness of the Velostat-based sensing element to create a 1.6 mm final sensor, which is the maximum possible sensing element thickness in our design. We did not increase the thickness beyond this 1.6 mm since a larger thickness would not be considered a textile sensor. This adjustment allowed us to extend this limitation to some extent, as the 1.6 mm sensor exhibited a linear voltage response up to approximately 610 N. It should be noted that the second-order polynomial line (blue) in Figure 8 was used to give some flexibility to fit the entire data range. This was only for visualization purposes and to demonstrate the trend beyond the threshold point. This second-order polynomial line also helps to determine the threshold points in the linear region.

In Figure 8a–c, linear regression lines (depicted in red) are also plotted with a 95% confidence interval, with data points with an upper threshold of 340 N, 420 N, and 610 N of the applied impact force for the 0.2 mm, 0.4 mm, and 1.6 mm sensors, respectively, being considered We used only linear regression lines to evaluate the sensor performance, such as its sensitivity. The corresponding linear equations, along with *m* and *b* values, are provided in the figure legends. In the linear area, the sensitivities of the 0.2 mm, 0.4 mm, and 1.6 mm STISs were 0.0197, 0.0106, and 0.0089 V/N, respectively. However, it was seen that at very large impact forces, no sensor showed a linear relation between force and voltage measurement, exhibiting saturation in their response. This phenomenon is primarily attributed to the deformation of the sensing materials resulting from powerful impacts. Despite not providing equivalent voltage readings reflective of the actual applied impact forces at high levels, the sensor may serve as an alarm for severe head injuries. Moreover, it can offer insights into the distribution of impact forces by measuring the places of the highest to lowest voltage, irrespective of the precise real values. From our experimental study, we found that the lower and upper threshold impact forces that the three sensors could detect were different. Hence, in Figure 8, the entire impact test range is not shown on the x-axis. In each subplot in Figure 8, the focus is on the maximum threshold force. Additionally, about an equal number of data points are in the left linear region and right saturation region of these threshold points. This was done to provide a fair comparison, to calculate the sensitivity, and to visualize the linear range.

**Figure 9:**
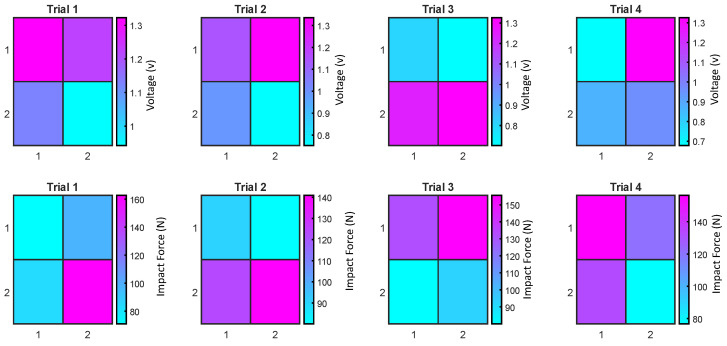
Voltage distribution in the first four tests (**first row**) and the corresponding impact force distribution (**second row**) in the experiment using the sensor with a 1.6 mm sensing element.

In Figure 9 (first row), the voltage distribution across the four sensor areas during the initial four tests on the 1.6 mm STIS is depicted. Figure 9 (second row) illustrates the estimated distribution of impact force in the four sensing areas during the same test. The voltage and impact force distributions reveal that impact did not consistently occur at the same location. This variability aligns with real-life scenarios despite our efforts to release the pendulum precisely from the same initial position. Perhaps the applied external force slightly changed the pendulum trajectory each time. It is worth noting that the STIS matrix can not only be helpful in measuring the amount of force but also provide impact location. As an illustration, in the first test, we observed the lowest voltage reading in the fourth sensor area, indicating a more concentrated impact in that specific region. For the second test, the impact appeared to be between the second and third areas. In the fourth test, the impact was distributed among the first, second, and third areas, suggesting that the impact was centered around the middle of the sensor, with a slight shift toward the first sensing area.

## 7. Discussion

In this study, a smart textile impact sensor with 2×2 sensing areas was developed and evaluated. The thickness of the sensing element was adjustable, and outcomes from three distinct thicknesses are discussed here. These sensors were incorporated into a microcontroller board. A pendulum-based experimental test setup was constructed to assess individual impact sensors on impacts. The results showed that this type of impact sensor is, in general, suitable for moderate impact levels, but as the impact force increases, the sensor becomes saturated and the sensor response becomes unreliable. Creating smart textile impact sensors for the high-impact ranges encountered in concussions is still challenging. By fabricating the sensor in a helmet with shock-absorbing material, the sensor may be able to operate within its operating range and be calibrated. Depending on the use and intended area of detection, the number and arrangement of sensors and supporting electronics can be embedded within a helmet. This research focused on maintaining sensor performance in the high-impact range. We sought to improve the sensors by increasing the semiconductive polymer composite (SPC) material thickness. Tests were repeated while the SPC thickness was gradually increased to detect higher impact forces. This study offers a comprehensive analysis of the sensor’s behavior and explores the impact of augmenting the sensing element’s thickness on its performance, specifically focusing on the linearity of the sensor.

Results showed the SPC-based impact sensors’ sensitivity drifted in the high impact range. Primarily, this phenomenon is attributed to the creep, or cold flow, observed in these composites. Creep refers to the gradual and long-term deformation of a solid material subjected to continuous high mechanical stress below the material’s yield strength [24]. This hysteresis effect results from the material’s inability to sustain its initial internal structure under intense impact. Certain studies on the performance of flexible impact sensors have reported enhanced detection ranges with various piezo-resistive materials. The reported detection ranges span from 0.2 Pa to 10 kPa, with materials being used including olydimethylsiloxane (PDMS) dispersed with conductive particles such as gold or carbon, aligned carbon nanotube (ACNT), and vertically aligned carbon nanotube (VACNT), among others. Notably, a significant improvement in detection ranges, reaching from 0.2 Pa to 59 kPa, has been achieved using an interlocked microdome array [25,26]. While this marks a crucial advancement, further enhancements are necessary to increase the maximum detection level and cover a broader range of concussions.

## 8. Conclusions and Future Work

Developing a reliable smart textile impact sensor for detecting high impacts using semiconductive polymer composites (SPCs) such as Velostat remains a formidable challenge. Addressing issues such as non-linearity, hysteresis, and repeatability is crucial for applications such as concussion detection. While increasing the SPC thickness expands the sensor range, significant thickness increments are needed to achieve noticeable changes. Robust testing methodologies beyond bench testing are essential to simulating real-world field scenarios and ensuring more trustworthy results. Overall, polymeric-based sensors exhibit subpar performance in the high range, yet their simplicity, cost-effectiveness, and wearability make them promising. Ongoing research focused on enhancing materials and structural designs holds potential for overcoming limitations and improving overall sensor performance. Although at extremely high impacts, the sensors’ readings did not reflect the actually applied impact forces, they did provides helpful information regarding the impact force distribution. They may also provide alerts for when impact force is beyond the threshold. The proposed smart textile impact sensor holds potential for future applications in electronic helmets (e-helmets). Further research and development in this domain could lead to modifications and enhancements that enable accurate measurement of concussion impact ranges. A significant stride in improvement could come from refining the structure of the sensing element or identifying alternative SPC materials capable of withstanding high impacts without compromising their internal structure, thereby enhancing the reliability of sensor readings.

The field of smart textiles is very exciting and promising. The present study is considered to be the beginning of more comprehensive studies to follow. Using more rigorous testing procedures, we will examine new combinations of materials and structures. We will conduct comprehensive studies, incorporating mathematical and computational modeling, to apply such sensors in real life. In this research, the tests were conducted to mimic real head-impact situations using a pendulum-based experimental test setup. Substantial enhancement of these sensors necessitates not just conducting laboratory bench tests with precise impact quantities but also conducting tests in authentic and on-field conditions. This approach is essential to gaining a more comprehensive understanding of potential real-world scenarios, ensuring the practical and secure deployment of this sensor type [16,24,27,28]. In the future, we will develop a testing protocol that would allow us to perform sensor testing on field. Furthermore, to enhance linearity, we will employ a suitable conditioning circuit. The utilization of a transimpedance amplifier is a viable solution, as it has the potential to enhance the sensitivity of a sensor to achieve closer linearity [24]. By implementing hardware enhancements and employing a polynomial equation, the calibration process can be further refined to enhance the sensor’s performance. In the future, an electronic helmet (e-Helmet) can be constructed with an STIS matrix that could cover the whole head within the helmet, and each sensor area could provide independent impact readings. Mapping these readings would provide a better and invaluable estimation of the state of head impact.

## Figures and Tables

**Table 1:** Commercial head impact measurement devices.

Device Name	Manufacturer	Technology Used	Location & Design
Brain Band	Samsung	Unspecified	Headband
Brain Sentry iC+	Brain Sentry	Unspecified	Outer back side of helmet
BodiTrak Head Health Network	Marucci Sports	Accelerometer, gyroscope, and thermometer and smart textile	In helmet
Checklight	Reebok	Accelerometer and gyroscope	Skullcap with or without helmet
FITGuard	Force Impact Technology	3-axis accelerometer, 3-axis angular rate sensor	Mouthguard
GForce Tracker	GForce Tracker	3-axis accelerometer and gyroscope	In helmet
Head Case	Head Case	Accelerometer	Mounted inside headgear of helmet
HeadsUp	Integrated Bionics, LLC	Unspecified	Headband or armband
Impact Assessment System	Linx	3-axis accelerometer and gyroscope	Headband or skullcap
Jolt Sensor	Jolt	Unspecified	On headband or helmet
PlayerMD	Archetype	6-degrees-of-freedom sensor array	Skullcap or headband
Rosh headband/cap	Rosh	4 impact sensors	Headband or full headcap
Riddell Sideline Response System	Riddell	6 single-axis accelerometers	In helmet
Riddell Insight Training Tool ITT	Riddell	5-zone sensor pad	In helmet
SIM-P (individual) SIM-G (team)	Triax	3-axis accelerometer and gyroscope	Headband or skullcap
Shockbox	i1 Biometrics	4 unidirectional, orthogonally placed force switches	Helmet
Vector	Vector	3-axis accelerometer and gyroscope	Mouthguard
X-Patch	X2 Biosystems	3-axis accelerometer and gyroscope	Behind ear
X-Guard	X2 Biosystems	3-axis accelerometer and gyroscope	Mouthguard

## Data Availability

Data are contained within the article.
